# Establishment of Cre/LoxP-mediated multifunctional reporter knock-in rats with the CRISPR system

**DOI:** 10.1371/journal.pone.0325444

**Published:** 2025-06-25

**Authors:** Katsuyuki Nakamura, Sara Ito, Yoshihiro Ohguchi, Toshie Jimbo, Yusaku Wada, Ryota Nakajima, Masanobu Kanou, Kei Yamana, Hiroshi Ueda

**Affiliations:** 1 Department of Chemistry and Biomolecular Science, Faculty of Engineering, Gifu University, Gifu, Japan; 2 Center for One Medicine Innovative Translational Research (COMIT), Institute for Advanced Study, Gifu University, Gifu, Japan; 3 Animal Management Group Bioscience Business Division, KAC Co., Ltd., Ritto, Shiga, Japan; 4 Teijin Ltd., Hino, Tokyo, Japan; 5 Research and Development Department, FASMAC Co., Ltd., Atsugi, Kanagawa, Japan; University of Minnesota Medical School, UNITED STATES OF AMERICA

## Abstract

Rats and mice are essential experimental animals in preclinical research, serving as models for various human diseases and contributing significantly to drug development. Rats offer distinct advantages over mice due to their larger size, which allows for more complex surgical procedures, repeated blood sampling, or sophisticated behavioral analysis. However, unlike the case with mice, genetically modified rat lines for achieving complex experimental objectives—such as tissue-specific gene knockout or visualization of specific cell populations—are still limited. We here established LoxP-mediated multifunctional reporter KI rats, enabling us to evaluate fluorescence, bioluminescence, and cell-killing assays simultaneously with only one gene-modified rat line. CRISPR/Cas12a, also known as CRISPR/Cpf1, was successfully used to insert the Cre sequence into a target locus to generate Cre driver rats. These results will contribute to the application of gene-modified rats for a more comprehensive understanding of physiology, and for extrapolation of their capabilities in preclinical research.

## Introduction

Like mice, rats are widely used in preclinical studies [1] as models for human diseases, providing significant insights that contribute to the advancement of biology [2,3]. Rats are more intelligent than mice and have the advantage of greater size for surgical experimental procedures and continuous blood sampling [4].

Moreover, rat genetic backgrounds have been shown to more accurately recapitulate human disease conditions compared to mice, aided by advancements in genome editing technologies such as CRISPR/Cas9 [5] and CRISPR/Cas12a (also known as CRISPR/Cpf1 [6]) to create gene-modified rats [7]. For example, mdx mice, a murine model of Duchenne muscular dystrophy (DMD), which is caused by a mutation in the Dmd gene in the X chromosome leading to progressive and degenerative muscle dysfunction, does not exhibit the severe, progressive, or degenerative pathologies seen in human DMD. In contrast, we previously generated DMD model rats using the CRISPR/Cas9 system [8], and found that their phenotypes were more severe than those of mice, and roughly equivalent to the pathology of human DMD [9,10]. Pompe disease model rats also exhibit a phenotype more akin to humans when compared to the murine model [11]. Even though research using knock-out (KO) or knock-in (KI) rats has become readily achievable [12,13], the opportunity to use gene-modified rats remains limited.

Gene-modifying experiments aiming for tissue-specific gene deletion with the Cre/LoxP system [14] have been conducted predominantly in mice [15]. These experiments involve detecting a target protein expression or specific cell populations using tags or fluorescent proteins, as well as eliminating specific cell populations using suicide genes such as HSV-TK [16] or diphtheria toxin and its receptor [17]. Double-fluorescence reporter rats with Cre/LoxP [18], luminescence reporter rats with firefly luciferase [19], and suicide gene‒expressing rats with thymidine kinase [20] have been established independently as transgenic lines without precise regulation of the locus or copy number of the transgene. The lineup of gene-modified rat strains such as Cre driver rats is also limited [21].

In this study we generated Cre/LoxP-mediated, multifunctional reporter KI rats by applying the CRISPR/Cas9 system to the rat Rosa26 gene locus [22]. This LoxP reporter rat will enable us to detect Cre expression by monitoring fluorescence changes from RFP to GFP, luminescence derived from NanoLuc, and the expression of thymidine kinase for cell-killing assay simultaneously with only one copy of the reporter gene in a specific target genome locus of the Rosa26 gene. Also, we demonstrated that Cpf1 can generate Cre driver KI rats by targeting a specific genome locus. These convenient tools will enable us to conduct more translational preclinical research by generating gene-modified rats, as previously done in mice.

## Materials and methods

### Generation of KI rats with CRISPR/Cas9 and Cpf1

Two guide RNAs (crRNAs, CACUCCCUGGAGUGCAAUGUguuuuagagcuaugcuguuuug and CAAGACCUACAUUGCACUCCguuuuagagcuaugcuguuuug), tracrRNA, Cas9 protein, and an HR vector were obtained from Fasmac (Kanagawa, Japan) and injected in a manner described previously [23]. Briefly, 30 IU of equine chorionic gonadotropin (eCG) was intraperitoneally injected into sexually immature female Wistar–Imamichi rats aged 8–11 weeks (Institute for Animal Production, Ibaraki, Japan). At 48 h after eCG injection, these rats were similarly injected with 30 IU of human chorionic gonadotropin and mated with male Wistar–Imamichi rats overnight. Superovulated zygotes were then removed from the oviducts of female rats, treated with hyaluronidase (Sigma-Aldrich, St. Louis, MO, USA), washed, and cultured in mR1ECM medium (ARK Resource, Kumamoto, Japan). They were then injected with 4 pL of a mixture of 100 ng/uL Cas9 protein, 50 ng/uL guide RNAs, and 5 ng/uL HR vector into the pronuclei of rat embryos with a micro-injector (Narishige, Tokyo, Japan).

For Cpf1 injection, the Cpf1 protein and two crRNAs (target regions: TACTTGGCTTTTCCACTTTCG and GGCTTTGTACTTGGCTTTTCC) were purchased from Integrated DNA Technologies, while ssDNA was manufactured by Fasmac (Kanagawa, Japan). A mixture of 50 ng/uL Cpf1 protein, 30 ng/uL of each of the crRNAs, and 50 ng/uL ssDNA was injected into the cytosol of rat embryos obtained by the method described above. The resulting zygotes were cultured in mR1ECM medium (ARK Resource, Kumamoto, Japan) for 1 h and transferred to the oviductal ampullae of pseudo-pregnant Wistar–Imamichi female rats.

For the first screening of *Rosa26*-reporter Tandem KI rats, two primer sets were used.

Primer set #1 (Forward: AATCAGAAGGGTGTATGGACTGCT; Reverse: ATACCCAGACCTCAGAAAGATAGGC)

Primer set #2 (Forward: GCGGATCAAAGAAGCGGATAAAGAA; Reverse: TAATTGTTCCAGTTGTTGCATCCCC).

For the first screening of *Rosa26*-reporter Flex KI rats, two primer sets were used:

Primer set #1 (Forward: GCCTTGATCACTTCCACAATGTTGA; Reverse: GCTGTTCGAGAAGGAGATTCCCTAT) Primer set #2 (Forward: GCGGATCAAAGAAGCGGATAAAGAA; Reverse: CGAGTAACCATCAACGGAGTGACC).

PCR for the confirmation of the Tandem and Flex KI alleles in the target region was conducted using the following primer sets. The first of these sets detected the sequence outside of the homologous arm; the second recognized a part of the KI allele to ensure that only KI rats, and not transgenic rats, were detected.

Primer Set #1 (F1: CCAGCAGTTGAAACTTTCCTTTAGA; R1: TTGTCCCAAATCTGTGCGGA)

Primer set #2 (F2: GCGTACTTGGCATATGATACACTTG; R2: ATACCCAGACCTCAGAAAGATAGGC).

For the confirmation of the vector insertion in Tandem and Flex KI alleles, PCR was conducted using the set of primers shown below.

Primer set for the Backbone (F: TATCAGATCGGAAGAGCAC; R: TATCAGATCGGAAGAGCAC)

Primer set for Ampicillin (F: GACTCCCCGTCGTGTAGATAACTAC; R: CGCTGGTGAAAGTAAAAGATGCTGA)

For the first screening of Cpf1-Cre KI rats, the primer set (F1: GCCCAGGAGCAGTTAGAAAG; R1: GGCGATCCCTGAACATGTC) was used.

For the confirmation of KI allele in the precise target region, the primer set (F2: CGCACTGATTTCGACCAGGTTC; R2: ATTCCACTGGGGAAACCTCT) was used.

PCR was performed using Gflex Tks DNA polymerase (Takara-Bio, Shiga, Japan) according to the manufacturer’s protocol. Briefly, 1.5 mL of each DNA isolated from the rat tail tip with 50 mM NaOH was added to 20 mL of the PCR mixture. After 38 cycles of the PCR reaction (extension time: 7 min for F1 + R1 and F2 + R2 in Tandem or Flex KI rats, 1.5 min for F1 + R1 and F2 + R2 in *Hprt1*-Cre KI rats and 3 min for other cases).

All animal experiments were approved by the Animal Care and Use Committee of the Bioscience Center of KAC Co., Ltd. and the Animal Care and Use Committee of Gifu University (approval no. AG-P-N-20240100). Rats were anesthetized for surgery with a combination of xylazine, butorphanol and diazepam. After recovery from the anesthetics, rats were warmed at 37°C with heat insulating materials. Before the experiments, rats were sacrificed by overdose of isoflurane.

### Isolation of fibroblasts from rat tail tips (rTTFs)

A 5 mm tail-tip segment was dissected from each 3-week-old F0 rat. For Tandem/Flex dKI rats, F1 rats were used for the isolation of rTTFs. The tips were minced and digested with collagenase type 2 (Worthington Biochemical, Lakewood, NJ, USA) and dispase 1 (FUJIFILM Wako, Tokyo) for 30 min at 37°C. The digested tissues were filtrated with a 40 mm cell strainer and centrifuged. The pellets were resuspended in DMEM with 10% FBS and plated on collagen-coated dishes. The following day, the media were refreshed, and the cells were incubated for subsequent experiments.

### Transfection of Cre mRNA

In vitro transcription of Cre mRNA was carried out by using an HiScribe T7 ARCA mRNA Kit (with Tailing, NEB, MA) according to the manufacturer’s protocol. Cre mRNA was transfected into rTTFs using Lipofectamine MessengerMAX (Invitrogen, Carlsbad, CA, USA). Nano-Glo® Vivazine™ Live Cell Substrates (N2580, Promega, Madison, WI, USA) or the Nano-Glo® Luciferase Assay System (N1110, Promega) was used to detect the luminescence of NanoLuc. In the cell-killing assay, GCV (TCI, Tokyo) was added to the cells. The luminescence signal was measured with Nivo S (PerkinElmer, Waltham, MA, USA).

### Immunostaining of rTTFs

Seven days after Cre recombination, rTTFs were fixed with 4% PFA in PBS for 15 min, then blocked with 5% NGS in PBS containing 0.1% Triton-X for 20 min. After blocking, the cells were treated with primary antibodies diluted in 5% NGS in PBS for 2 h at room temperature. The treated cells were then washed and stained with the diluted secondary antibodies. The primary antibody used in this study was anti-cometGFP (1:2000, rabbit polyclonal; ATUM, Newark, CA, USA). The nuclei were counterstained with DAPI. The fluorescent images were captured using a BZX-810 microscope (Keyence, Osaka, Japan).

### Flow cytometry

The cometGFP and fresnoRFP signals of the WT, Tandem, Flex, and Tandem/Flex rTTFs after transfecting Cre mRNA were analyzed using a cell sorter (SH800, Sony, Tokyo). The graph was generated using FlowJo (BD Biosciences, Franklin Lakes, NJ, USA).

### Statistical analysis

Differences between the WT rats and the KI rats with or without Cre mRNA transfection were evaluated by two-way ANOVA followed by multiple comparisons using the Šidák multiple comparisons test. In addition, differences within a single group (Fig 2H) were detected using one-way ANOVA followed by Dunnett’s multiple comparisons test. The numbers of samples (means ± SD) are indicated in the graphs. P values lower than 0.05 were considered statistically significant. GraphPad Prism10 software was used to generate all graphs and conduct all statistical analyses.

## Results

### Generation of Tandem LoxP *Rosa26*-reporter KI rats with CRISPR/Cas9

**Fig 1:**
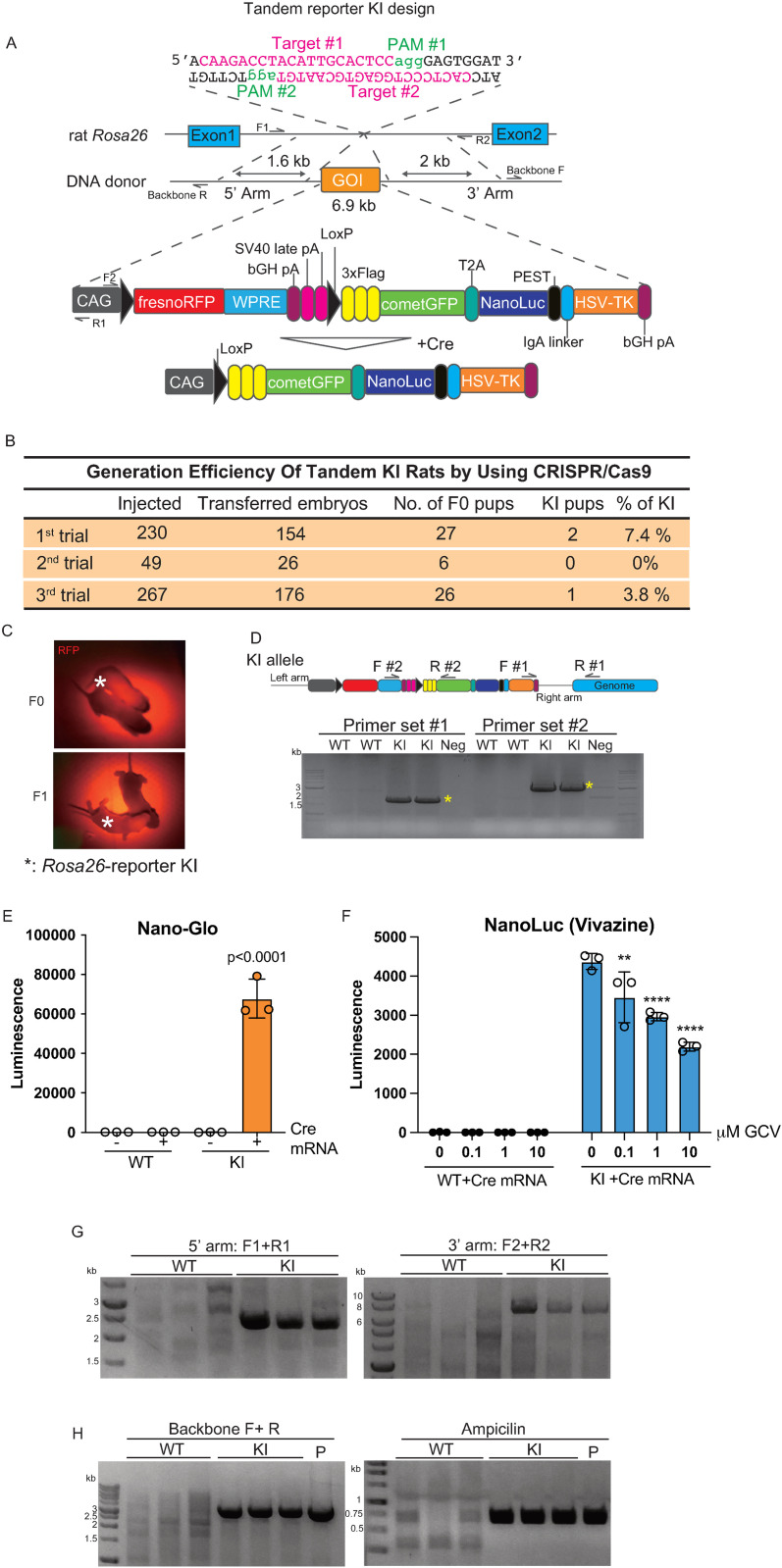
Generation of LoxP multifunctional Cre/LoxP reporter knock-in (KI) rats with CRISPR/Cas9. A: Schematic representation of a LoxP multifunctional reporter KI rat at the Rosa26 gene locus using the CRISPR/Cas9 system. PAM sequences are depicted in green, and target sequences are shown in magenta. B: Efficiency of the generation of *Rosa26*-reporter KI rats using CRISPR/Cas9. C: Representative fluorescent images of F0 and F1 pups of *Rosa26*-reporter KI rats. D: Representative images of genotyping PCR for Tandem KI rats. Primer sets are indicated in the KI allele scheme. E: Luminescence of NanoLuc in rTTFs from WT or *Rosa26*-reporter KI rats with or without Cre mRNA transfection. Data are represented as mean ± standard deviation (SD) of triplicate experiments. P-values were determined by two-way ANOVA followed by Šidák multiple comparisons test. F: Cell-killing assay in rTTFs from WT or *Rosa26*-reporter KI rats after Cre mRNA transfection with GCV. The NanoLuc signal was assessed with Vivazine. Data are represented as mean ± standard deviation (SD) of triplicate experiments. **: p < 0.01, ****: p < 0.0001 vs 0 mM GCV (two-way ANOVA followed by Šidák multiple comparisons test). G: The results of PCR from the outside of the HR arm to the inside of the Tandem reporter KI cassette at the 5’ and 3’ arms. The primers used in this PCR are indicated in the KI scheme of Fig 1A. H: The PCR results from the genome of Tandem KI rats to confirm the insertion of the full length of the backbone (Backbone) and the resistance gene (Ampicillin). P: Tandem KI vector as a positive control of the PCR.

To achieve multiple experimental objectives efficiently, we knocked in a floxed RFP with polyA signals followed by GFP to detect the target cell population, NanoLuc luminescence for screening and validating autofluorescence, and Herpes simplex virus thymidine kinase (HSV-TK) for the cell-killing assay into the rat Rosa26 gene locus with Cas9 and two guide RNAs (Fig 1A; referred to as Tandem-version rats hereinafter [24]). We obtained several *Rosa26*-reporter KI F0 pups confirmed by PCR using two different primer sets (Fig 1B and 1C). RFP signals of the Tandem reporter KI rats were detectable in both F0 and F1 pups (Fig 1D), indicating germline transmission of the KI allele.

Next, we established cell lines from the fibroblasts isolated from the KI rat tail tips (rTTFs) to assess the expression of reporter genes before and after Cre recombination in vitro. In the Tandem KI design, the GFP signal strength after Cre recombination in rTTFs was not sufficient to be visible without immunostaining under a fluorescence microscope. The NanoLuc signal was detected in the Tandem KI rTTFs but not in the WT rTTFs or the rTTFs of the KI rats without Cre mRNA transfection (Fig 1E). The cell-killing assay performed with ganciclovir (GCV), followed by NanoLuc-based detection of cell numbers, showed a dose-dependent decrease in the number of KI rTTFs expressing HSV-TK (Fig 1F). After the F1 generation, PCR analysis with the primers recognizing the region from recognizing from the outside of the HR arm to the inside of the KI cassette at both the 5’ and 3’ arms confirmed that these reporter genes were inserted in the precise region targeted by CRISPR/Cas9 (Fig 1G), albeit with unexpected insertion of the full length of the backbone vector (Fig 1G and 1H). These results demonstrate the establishment of multifunctional LoxP-mediated *Rosa26*-reporter KI rats whose reporter expression relies on Cre recombinase activity.

### Generation of Flex LoxP *Rosa26*-reporter KI rats with CRISPR/Cas9

**Fig 2:**
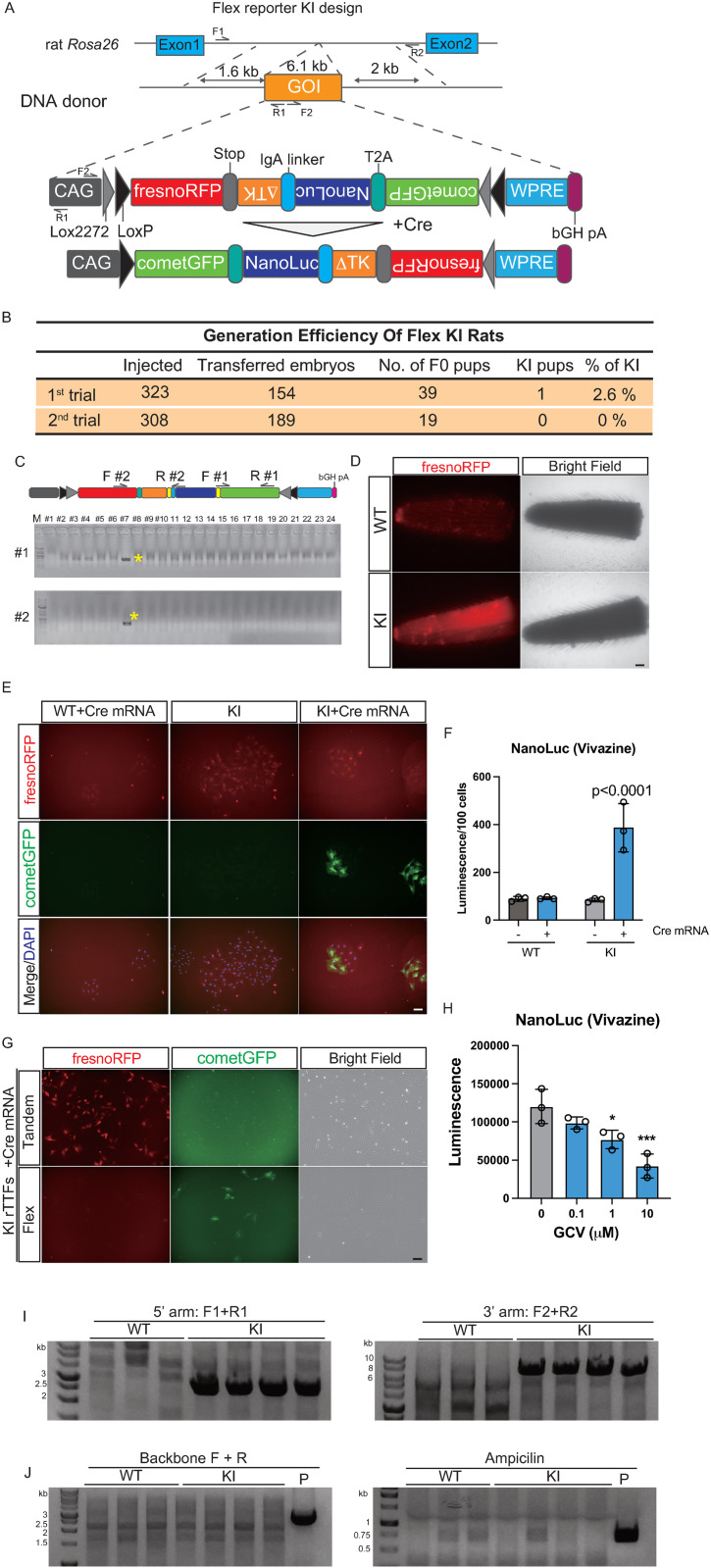
Generation of Flex *Rosa26*-reporter KI rats with the CRISPR/Cas9. A: Schematic representation of the Flex version of *Rosa26*-reporter KI rats (Flex KI rats) at the Rosa26 gene locus with CRISPR/Cas9 systems. B: Efficiency of the generation of Flex *Rosa26*-reporter KI rats. C: Representative fluorescent images of tail tips from WT and KI rats. Scale bar = 1 mm. D: Representative images of genotyping PCR of Flex KI rats. Primer sets are indicated in the KI allele scheme. E: Immunostaining of cometGFP in rTTFs from WT or Flex KI rats with or without Cre mRNA transfection. Scale bar = 100 μm. F: Luminescence of NanoLuc in rTTFs from WT or Flex KI rats with or without Cre mRNA transfection. Data are represented as mean ± SD of triplicate experiments. P-values were determined by two-way ANOVA followed by Šidák multiple comparisons test. G: Fluorescent images of Tandem or Flex KI rTTFs. Scale bar = 100 μm. H: Cell-killing assay of the GCV treatment in Flex KI rTTFs assessed with the luminescence of NanoLuc. Data are represented as mean ± SD of triplicate experiments. *: p < 0.05, ***: p < 0.001 (one-way ANOVA followed by Dunnett’s multiple comparisons test). I: The results of PCR from the outside of the HR arm to the inside of the Flex reporter KI cassette at the 5’ and 3’ arms. The primers used in this PCR are indicated in the KI scheme of Fig 2A. J: The PCR results from the genome of Flex KI rats to confirm insertion of the full-length backbone (Backbone) and the resistance gene (Ampicillin). P: Flex KI vector as a positive control of the PCR.

To enhance the GFP signal in Tandem KI rats after Cre recombination, we applied the Flex system to incorporate a single WPRE sequence for both RFP and GFP fluorescence (Fig 2A; Flex-version rats hereinafter). F0 pups carrying the KI allele, as confirmed by PCR using two different primer sets, were obtained (Fig 2B and 2C). The RFP signal from the tail tip of the Flex KI rat was detected under a standard fluorescence microscope (Fig 2D). To evaluate the expression of reporter genes in Flex KI rats, fibroblasts were isolated from the tail tips of Flex KI rats and transfected with Cre mRNA. The GFP signal, along with immunostaining and NanoLuc luminescence, was observed in Flex KI rTTFs with Cre mRNA specifically (Fig 2E and 2F). RFP and GFP signals without immunostaining were directly compared between Tandem and Flex KI rTTFs (Fig 2G). The RFP signal was attenuated in Flex compared to Tandem KI rTTFs, whereas the GFP signal was stronger in the Flex than in the Tandem KI rTTFs. After the F1 generation, PCR analysis from the outside of the HR arm to the inside of the KI cassette at both the 5’ and 3’ HR arms confirmed that these reporter genes were inserted precisely in the target locus, without insertion of the backbone vector (Fig 2I and 2J). These results indicate that while the Flex design does not sustain the robust RFP signal observed in the Tandem KI design, the Flex KI design does enhance the attenuated GFP signal in the Tandem KI design after Cre recombination.

### Combination of Tandem and Flex *Rosa26*-reporter KI rats

**Fig 3:**
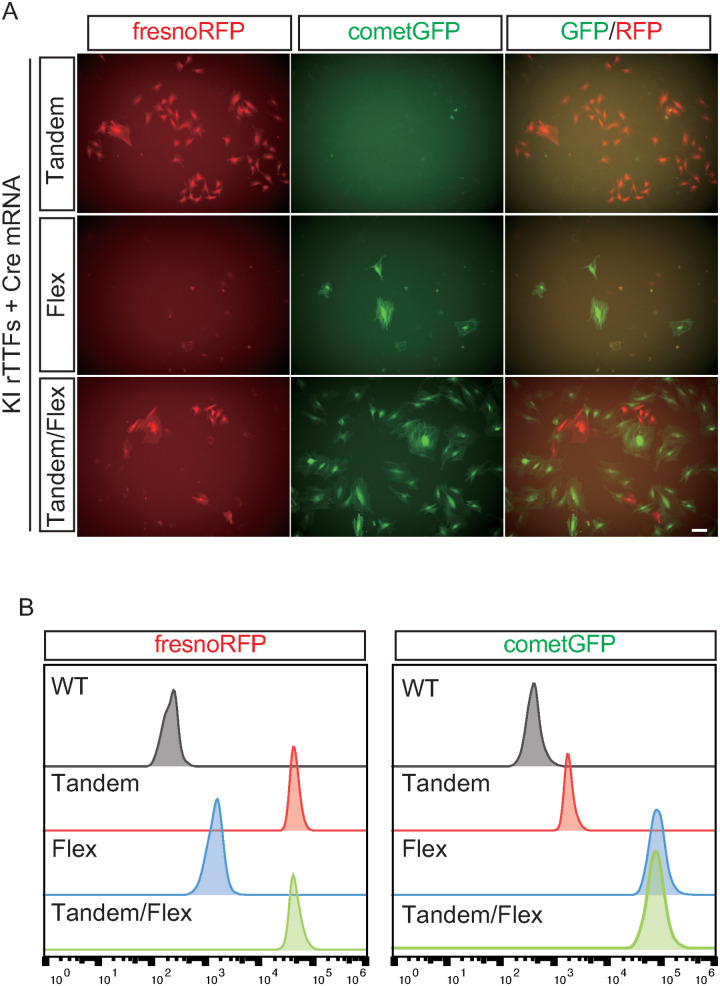
Combination of Tandem and Flex KI rats to enhance both RFP and GFP signals. A: Representative fluorescence images of rTTFs from Tandem, Flex, and Tandem/Flex KI rats. Scale bar = 100 μm. B: Flow cytometry analysis of cometGFP or fresnoRFP signals in rTTFs from Tandem, Flex, and Tandem/Flex KI rats.

To attain a robust RFP signaling prior to Cre recombination simultaneously with a strong GFP signal after Cre recombination, we merged the Tandem and Flex KI designs, aiming to leverage the strengths of both approaches. Tandem and Flex KI rats were crossed, and the fluorescence intensity in rTTFs from Tandem, Flex, or Tandem/Flex KI rats was assessed microscopically (Fig 3A). The crossing of the Tandem and Flex KI designs enhanced the weakened RFP signal in the Flex KI design before Cre recombination, while also improving the attenuated GFP signal of the Tandem KI design after Cre recombination. These findings were consistent with those obtained through flow cytometry analysis (Fig 3B). Therefore, the simultaneous application of the Tandem and Flex KI designs was shown to produce robust RFP and GFP signals before and after Cre recombination, offering a promising strategy for the fluorescence-based reporting systems.

### Generation of *Hprt1*-Cre KI rats with CRISPR/Cas12a, Cpf1

**Fig 4:**
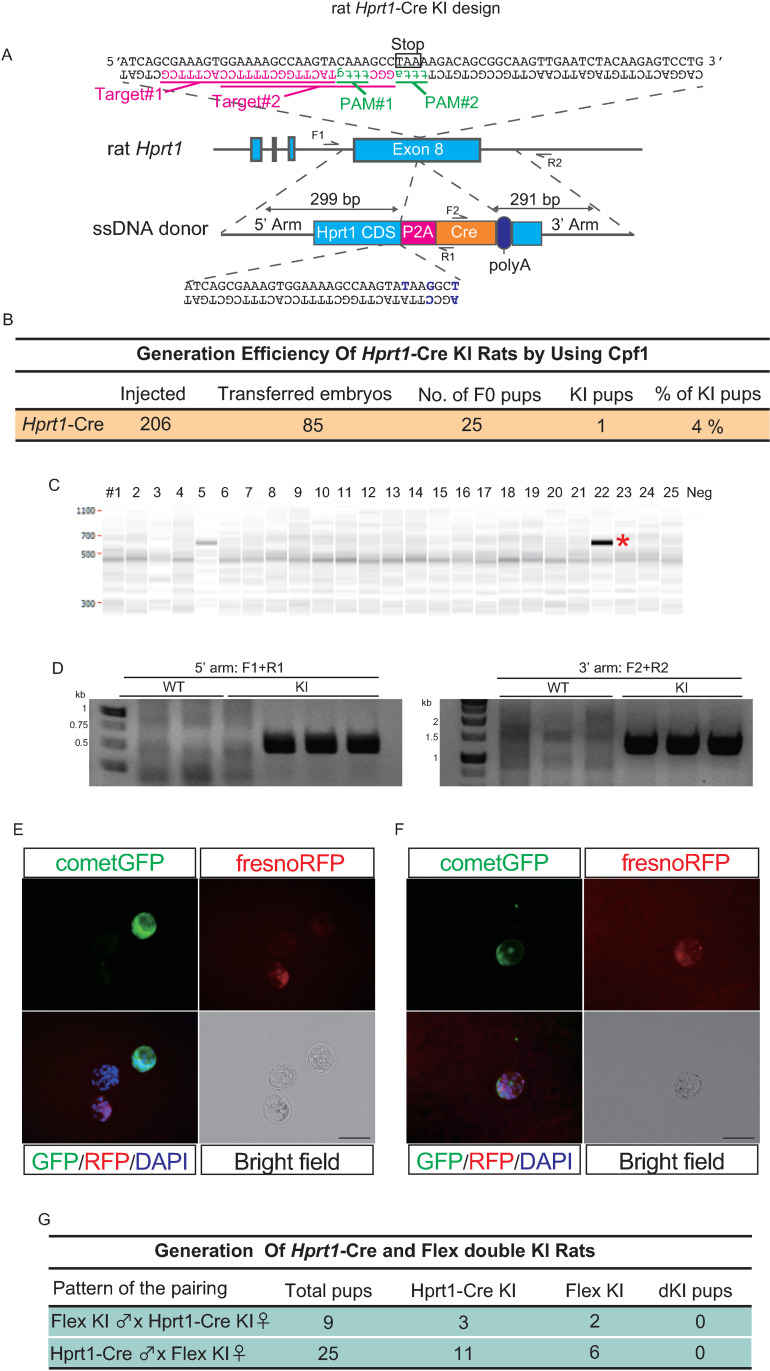
Generation of *Hprt1*-Cre KI rats with Cpf1. A: Schematic representation of Cre KI rats at the Hprt1 gene locus using Cpf1. PAM sequences are shown in green, and target sequences are shown in magenta. The bases changed in the donor ssDNA to prevent repeated clearance by Cpf1 after homologous recombination are shown in blue. B: Efficiency of the generation of *Hprt1*-Cre KI rats using Cpf1. C: Representative images of genotyping PCR for *Hprt1*-Cre KI rats. The primer sets are indicated in Fig 4A. D: The results of PCR analysis from the outside of the HR arm to the inside of the *Hprt1*-Cre KI cassette at the 5’ and 3’ arms. The primers used in this PCR are indicated in Fig 4A. E and F: Immunostaining of rat blastocysts after crossing *Hprt1*-Cre KI and Flex KI rats. Scale bar = 100 μm. G: Efficiency of the generation of *Hprt1*-Cre KI and Flex double KI rats.

Finally, we sought to confirm the transition of fluorescence signals from RFP to GFP in Flex KI rats by crossing them with Cre driver rats. To establish a Cre driver rat, Cpf1 was employed to insert the KI Cre sequence into the rat Hprt1 gene region; this insertion was designed to maintain endogenous *Hprt1* expression by placing a 2A peptide between the stop codon of the Hprt1 gene and Cre cDNA (Fig 4A). To prevent inadvertent Cpf1-mediated editing of the target site after recombination, three silent mutations were introduced into the homologous recombination vector by changing the sequence of the crRNA binding sites and their PAM sequences. Genotyping PCR of the F0 pups revealed the presence of one KI pup in the F0 generation (Fig 4B and 4C). After F1 generation, PCR analyses from the outside of the HR arm to the inside of the KI cassette at both the 5’ and 3’ HR arms were conducted and confirmed that the Cre sequence was inserted in the target locus (Fig 4D). Blastocysts collected from the double KI rats resulting from a cross between the Flex KI rats and *Hprt1*-Cre KI rats exhibited a transition from the RFP signal to the GFP signal (Fig 4E). A double-positive blastocyst with concurrent RFP-positive cells and GFP-positive cells was also observed, possibly resulting from random inactivation of the X chromosome where the Hprt1 gene is coded (Fig 4F). To date, our crossing has not yielded any double KI pups (Fig 4G), suggesting potential toxicity of the reporter genes in the developmental stage, leading to embryonic lethality (see the Discussion for details). These results suggest that while regulating the expression of Cre recombinase may be necessary in a tissue- or time-specific manner, the Flex reporter system can function effectively after crossing Cre driver rats.

## Discussion

The genome engineering tools of Cas9 and Cpf1 have been developed not only as experimental tools for the establishment of disease model animals and cell lines, but also as gene therapeutic options for genetic disorders [25], and as biosensors for viruses or cancer diagnosis [26]. We established LoxP-mediated multifunctional reporter KI rats that allowed us to simultaneously conduct fluorescence, bioluminescence, and cell-killing assays using only a single gene-modified rat line. In addition, we established *Hprt1*-Cre KI rats by using Cpf1, another type of CRISPR system. Cpf1 has already been used to create KI mice [27,28] and KO rats [29]. To the best of our knowledge, however, this study is the first to show that Cpf1 is applicable for KI of a long sequence like Cre into the target genome locus in rats. The PAM sequence of Cpf1 (TTTV) is different from that of Cas9 (NGG). Adapting the codon reading frame by conjugation with the 2A peptide is often mandatory for the generation of Cre or reporter KI rats with preserved endogenous expression of the target gene. Increasing the options for choosing the target sites of gRNAs from Cas9 or Cpf1 will be useful for achieving polycistronic expression of the inserted reporters and the effective production of KI rats.

Tandem KI rats showed weak GFP expression after Cre recombination. In the Tandem KI design, mRNA expressed from the GFP cassette after Cre recombination is not stabilized by WPRE. To improve this attenuated GFP signal in the Tandem design, WPRE sequence was applied for both RFP and GFP fluorescence in the Flex KI design. As shown in Figs 2 and 3, the GFP signal after Cre recombination was improved enough to be detected without immunostaining. However, the RFP signal before Cre recombination became weak. The length of the mRNA-containing RFP sequence was greater in the Flex KI design than in the Tandem KI design, which may have worsened the efficacy of RFP expression in the Flex KI design.

Although to our knowledge no studies have directly addressed the toxicity of systemic HSV-TK expression, it is known that HSV-TK causes an imbalance of the dNTP pool and cell death in human iPS cells [30]. This suggests that the ubiquitous expression of HSV-TK under a strong CAG promoter following systemic Cre expression originating from *Hprt1* may have contributed to developmental abnormalities or embryonic lethality. This, in turn, may explain why we have not obtained any *Hprt1*-Cre and Flex double-KI rats so far. To prevent this embryonic lethality, the reporters used in the KI rats generated in this study must be regulated to avoid expression during the developmental stages. Injection of the virus containing the expression cassette of Cre is one of the options to limit the Cre expression after birth. Another scenario for using the reporter KI rats established in this study would be achieved by a spatiotemporal Cre expression of genes with *CreERT2*, a Tamoxifen-induced Cre recombination coupled with Cre fused with the estrogen receptor, together with a tissue-specific promoter to restrict Cre expression solely to the target cells, such as *Pax7*, a marker of skeletal muscle stem cells [31]. Autofluorescence is known to cause significant difficulties for the immunohistochemical analysis of degenerated tissues using a fluorescence dye. In the KI rats generated in this study, Cre-expressing cells could be detected not only by the expression of the green fluorescent protein, as confirmed by bioluminescence with a low background compared to the fluorescence signal, but also by the diminishment of red fluorescence. These approaches can overcome the embryonic toxicity of Tandem/Flex dKI rats and allow us to report the target cells in disease model rats, such as DMD rats, which may better replicate the human condition than the corresponding models in mice.

This study has limitations. No double-KI pup of *Hprt1*-Cre/Flex *Rosa26*-reporter KI rats was obtained. This absence may be attributable to the strong expression of truncated HSV-TK, delta TK, controlled by the CAG promoter in the Flex *Rosa26*-reporter KI cassette after Cre recombination. Notably, HSV-TK can be expressed ectopically in the testis due to the cryptic promoter activity of the 5’ region of its cDNA, leading to toxicity in spermatogenesis and resulting in male infertility, a well-known phenomenon in transgenic animals [32,33].

Another limitation of this study is that off-target effects were not assessed. Cpf1 has been reported to have high specificity and lower off-target effects than Cas9 [34], but another group published data showing that Cpf1 has the potential to cause off-target effects [35]. For future analyses using the KI rats generated in this study, it will be necessary to lower the potential risk of off-target effects through several cycles of back-crossing of these KI rats with WT rats and selecting only KI rats from the pups.

In conclusion, we generated Cre/LoxP-mediated, multifunctional reporter KI rats using the CRISPR/Cas9 system at the rat Rosa26 gene locus. In addition, we demonstrated the utility of Cpf1 for creating Cre driver KI rats by modification at the target genome locus. These results move translational preclinical research employing gene-modified rats closer to that conducted in mice, and should promote the use of gene-modified rats for a more comprehensive understanding of physiology and for extrapolation of their capabilities in preclinical research.

## Supporting information

S1 Raw images Raw agarose gel electrophoresis images of the PCR products used in this study. (PDF)

## Abbreviations

KI: knock-in
HSV-TK: Herpes simplex virus thymidine kinase
GCV: ganciclovir
WPRE: woodchuck hepatitis virus posttranscriptional regulatory element
rTTFs: rat tail-tip‒derived fibroblasts
HR: homologous recombination
DMD: Duchenne muscular dystrophy
*Hprt1*: hypoxanthine phosphoribosyltransferase 1
