# Supplementary figures and images for: Establishment of Cre/LoxP-mediated multifunctional reporter knock-in rats with the CRISPR system
Source: PLoS One. 2025 Jun 25;20(6):e0325444. doi: 10.1371/journal.pone.0325444 (PMC12192107; doi:10.1371/journal.pone.0325444)

Fig. 1D

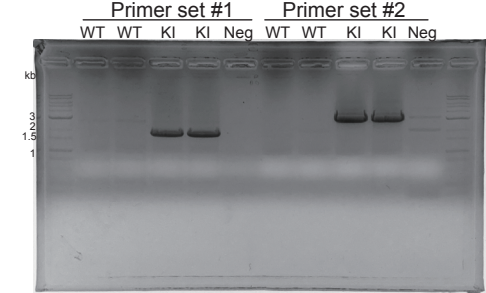

Fig. 1G

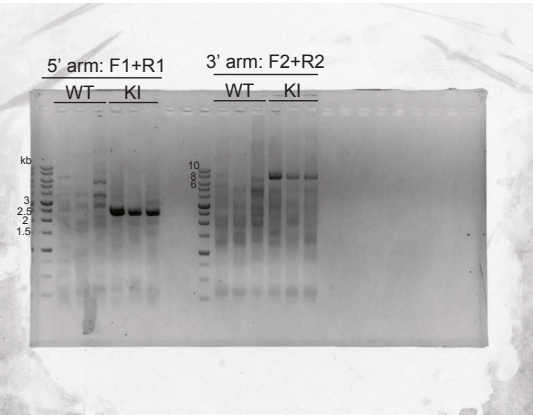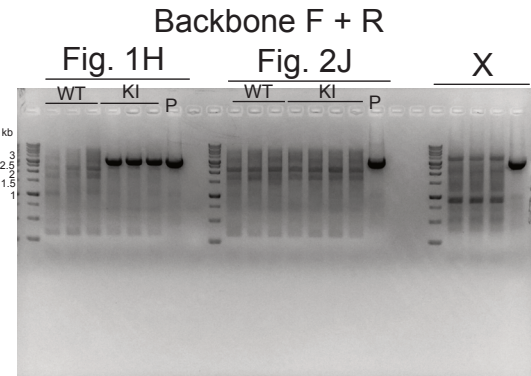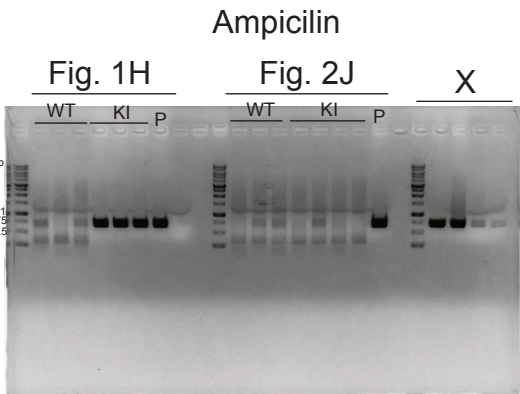

Fig. 2C

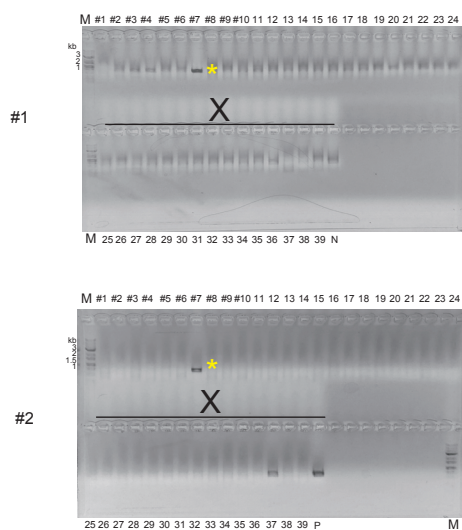

Fig. 2I

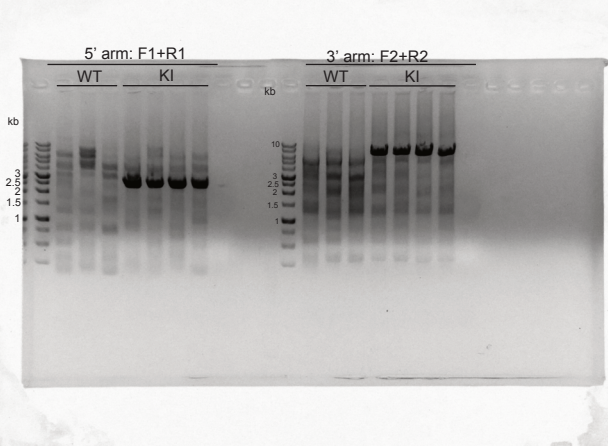

Fig. 4C

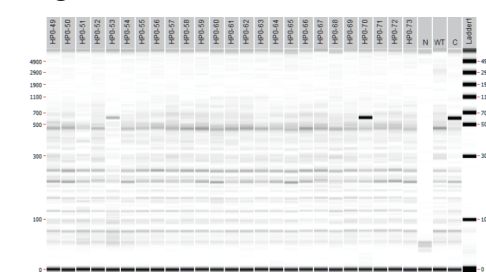

Fig. 4D

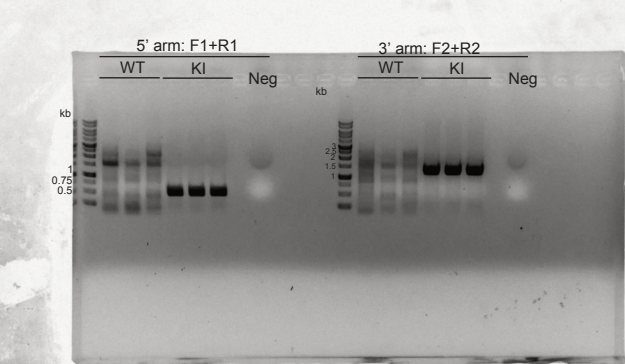

Supplement: S1 Raw images — (PDF) [file pone.0325444.s001.pdf]
